# Supplementary material for: Deep Learning Insights into the Dynamic Effects of Photodynamic Therapy on Cancer Cells
Source: Pharmaceutics. 2024 May 16;16(5):673. doi: 10.3390/pharmaceutics16050673 (PMC11125085; doi:10.3390/pharmaceutics16050673)
Supplement: Supplementary file 1 [file pharmaceutics-16-00673-s001.zip › pharmaceutics-2968244-supplementary.pdf]

Supplementary Information

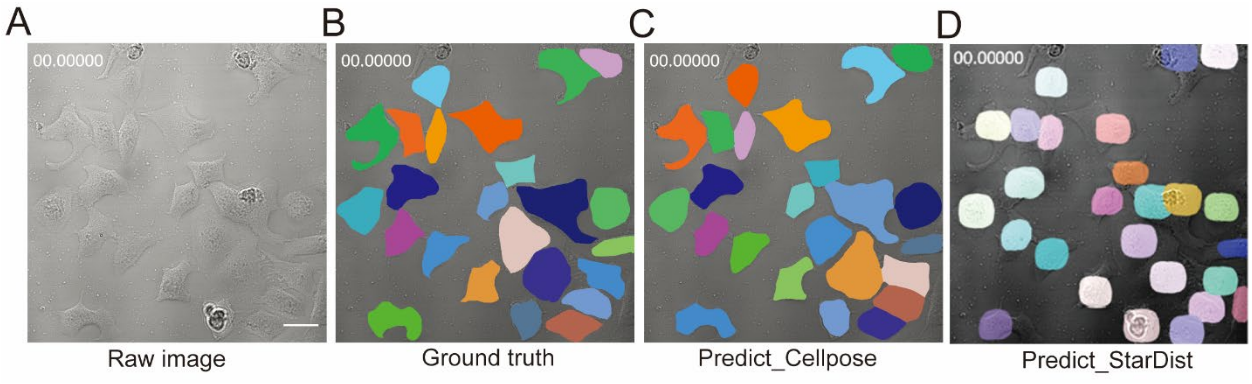

Figure S1. The Cellpose and StarDist models are compared to a highly accurate reference dataset (ground truth) to assess their validation. (**A**) The raw cell image. Scale bar: 30 μm. (**B**) The precise ground truth cell segmentation. (**C**) The Cellpose model's successfully segmented cells while maintaining their original morphologies. (**D**) The StarDist exhibited a propensity to predict primarily round shapes, even for cells with demonstrably different morphologies in the ground truth data.
